# Treatment reduces the incidence of newly appearing multiple sclerosis lesions evolving into chronic active, slowly expanding lesions: A retrospective analysis

**DOI:** 10.1111/ene.16092

**Published:** 2023-10-12

**Authors:** Alberto Calvi, Zoe Mendelsohn, Weaam Hamed, Declan Chard, Carmen Tur, Jon Stutters, David MacManus, Baris Kanber, Claudia A. M. Gandini Wheeler‐Kingshott, Frederik Barkhof, Ferran Prados

**Affiliations:** ^1^ NMR Research Unit, Institute of Neurology University College London London UK; ^2^ Laboratory of Advanced Imaging in Neuroimmunological Diseases, Hospital Clinic Barcelona, Fundació Clinic per a la Recerca Biomèdica Barcelona Spain; ^3^ Department of Radiology Charité School of Medicine and University Hospital Berlin Berlin Germany; ^4^ Department of Radiology Mansoura University Hospital Mansoura Egypt; ^5^ National Institute for Health Research, Biomedical Research Centre University College London Hospitals London UK; ^6^ Neurology‐Neuroimmunology Department Multiple Sclerosis Centre of Catalonia, Vall d'Hebron Barcelona Hospital Campus Barcelona Spain; ^7^ Department of Medical Physics and Biomedical Engineering, Centre for Medical Image Computing University College London London UK; ^8^ Department of Brain and Behavioral Sciences University of Pavia Pavia Italy; ^9^ Radiology and Nuclear Medicine, Amsterdam University Medical Centers (UMC) Vrije Universiteit Amsterdam the Netherlands; ^10^ e‐Health Centre Universitat Oberta de Catalunya Barcelona Spain

**Keywords:** chronic active lesions, fingolimod, primary progressive multiple sclerosis, slowly expanding lesions (SELs), volumetric MRI

## Abstract

**Background and purpose:**

Newly appearing lesions in multiple sclerosis (MS) may evolve into chronically active, slowly expanding lesions (SELs), leading to sustained disability progression. The aim of this study was to evaluate the incidence of newly appearing lesions developing into SELs, and their correlation to clinical evolution and treatment.

**Methods:**

A retrospective analysis of a fingolimod trial in primary progressive MS (PPMS; INFORMS, NCT 00731692) was undertaken. Data were available from 324 patients with magnetic resonance imaging scans up to 3 years after screening. New lesions at year 1 were identified with convolutional neural networks, and SELs obtained through a deformation‐based method. Clinical disability was assessed annually by Expanded Disability Status Scale (EDSS), Nine‐Hole Peg Test, Timed 25‐Foot Walk, and Paced Auditory Serial Addition Test. Linear, logistic, and mixed‐effect models were used to assess the relationship between the Jacobian expansion in new lesions and SELs, disability scores, and treatment status.

**Results:**

One hundred seventy patients had ≥1 new lesions at year 1 and had a higher lesion count at screening compared to patients with no new lesions (median = 27 vs. 22, *p* = 0.007). Among the new lesions (median = 2 per patient), 37% evolved into definite or possible SELs. Higher SEL volume and count were associated with EDSS worsening and confirmed disability progression. Treated patients had lower volume and count of definite SELs (*β* = −0.04, 95% confidence interval [CI] = −0.07 to −0.01, *p* = 0.015; *β* = −0.36, 95% CI = −0.67 to −0.06, *p* = 0.019, respectively).

**Conclusions:**

Incident chronic active lesions are common in PPMS, and fingolimod treatment can reduce their number.

## INTRODUCTION

Primary progressive multiple sclerosis (PPMS) is characterized by neurological deterioration without clinically evident neurological relapses, also recently defined as progression independent of relapse (PIRA) [1]. In relapse‐onset multiple sclerosis (MS), new demyelinating lesions are associated with clinical relapses [2]. The formation of new lesions is not closely linked with disability progression in PPMS [3], which is characterized by a lower load of new lesions on follow‐up magnetic resonance imaging (MRI) [4]. Instead, in PPMS, the accumulation of chronic active lesions appears to be more relevant [5, 6]. Chronically active lesions can be identified on MRI by lesion expansion (such lesions are termed *slowly expanding lesions* [SELs] [7, 8]).

Greater fraction of SELs compared with non‐SELs, has been associated with disability progression in both progressive and relapsing‐onset MS [9, 10]. It is estimated that 12% [9] to 29% [11] of the total lesion burden in all MS clinical phenotypes are SELs. The MRI quantitative features of SELs are consistent with the neuroaxonal damage observed in chronic active lesions and measured as T1 hypointensities [12], reduced magnetization transfer ratio (MTR) [11, 13], and increased radial diffusivity on diffusion‐weighted imaging [10, 14, 15]. SELs were recently found to correlate with other pathologically relevant markers of chronic active lesions, namely paramagnetic rim lesions, and their colocalization might represent the most destructive type of chronic MS lesions [16, 17].

In studies published so far, chronic expansion has only been assessed in (prevalent) lesions present at baseline, and therefore the impact of treatment on new (incident) lesions is unclear. To address this knowledge gap, we examined the impact of treatment on new lesions on follow‐up MRI using data from the INFORMS trial assessing the impact of fingolimod in PPMS.

The objectives were (1) to evaluate the number and volumes of incident lesions evolving into SELs, (2) to investigate the effect of fingolimod treatment on SELs, and (3) to assess the effect of incident SELs on clinical evolution.

## METHODS

### Inclusion criteria

**FIGURE 1:**
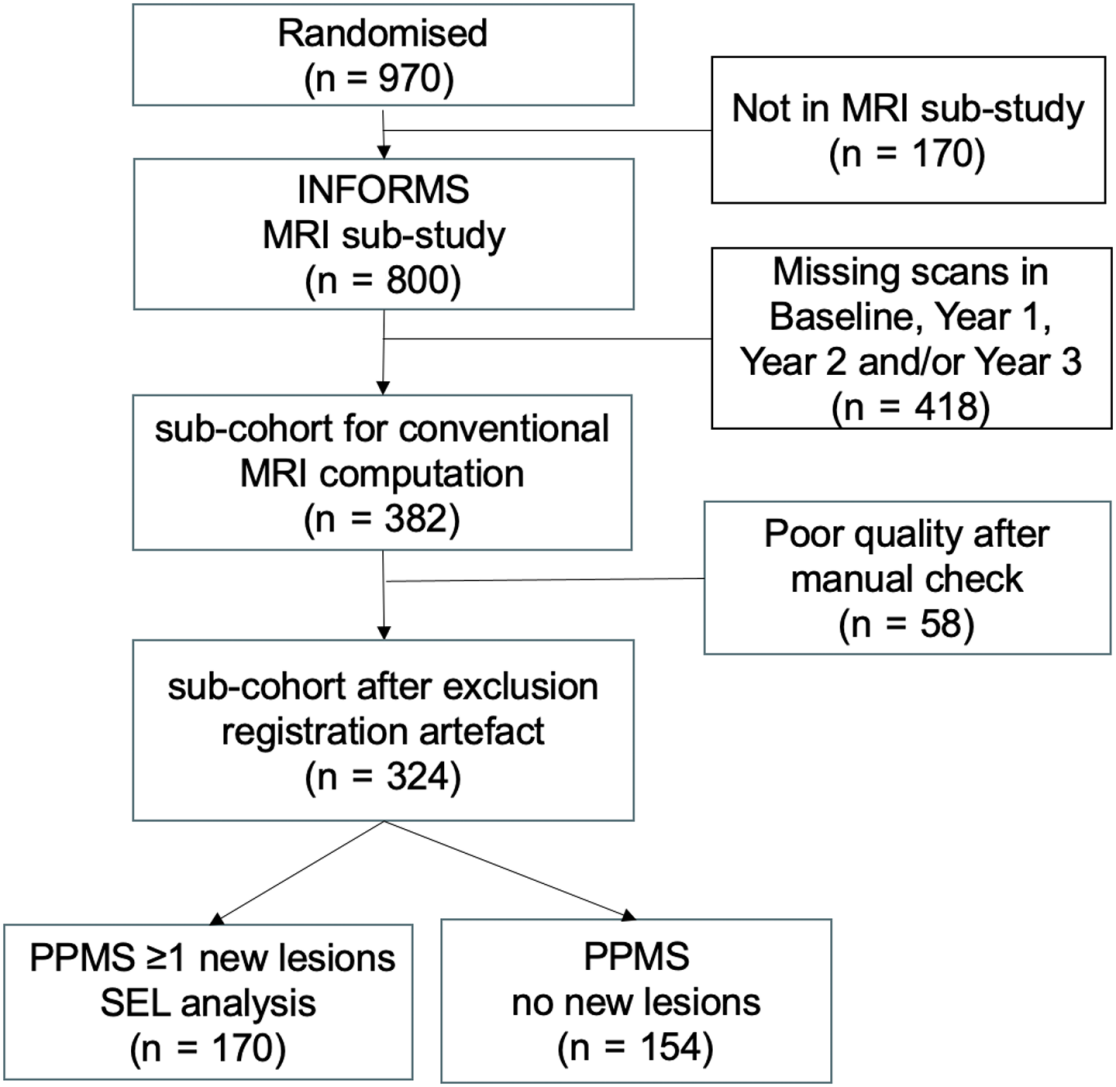
Flowchart showing the enrolment of subjects into the study. MRI, magnetic resonance imaging; PPMS, primary progressive multiple sclerosis; SEL, slowly expanding lesion.

We performed a retrospective analysis of the INFORMS trial data (NCT 00731692), a multicentre, double‐blind, placebo‐controlled, parallel‐group study assessing the efficacy of fingolimod in PPMS [18]. At trial recruitment, the investigators enrolled people aged 25–65 years with a clinical diagnosis of PPMS according to the 2005 revised McDonald criteria and additional conditions as previously explained [18]. Of 970 patients initially enrolled between 3 September 2008 and 30 August 2011, 800 had MRI scans available (INFORMS MRI substudy). Data were provided under the agreement of the International Progressive MS Alliance (IPMSA), and the institutional review board of the Montreal Neurological Institute (MNI), Quebec, Canada approved this study (reference number: IRB00010120). For this analysis, the following inclusion criteria were used: availability of (1) clinical data and (2) both T1‐weighted and fluid‐attenuated inversion recovery (FLAIR) images up to at least 3 years after screening. A total of 418 patients were not included due to missing scans at baseline, year 1, and/or year 3. This results in a cohort of 382 patients who underwent tissue segmentation. The artefacts affecting segmentation and registration led to the further exclusion of 58 patients. Of the remaining 324, 170 patients had one or more new lesions at year 1. These patients were defined as “PPMS ≥1 new lesions” and were used in the SEL analysis outlined below. The remaining PPMS patients did not have new lesions and were defined as “PPMS no new lesions”. The inclusion flow chart is shown in Figure 1.

### Data collection and clinical assessments

Written informed consent was obtained from all subjects, and the study protocol was approved by each site's institutional review board as detailed in the research trial publication [18]. Fully anonymized clinical and MRI data were analysed at Queen Square MS Centre, University College London. Patients were randomly assigned (1:1) to receive fingolimod 0.5 mg/1.25 mg (and after 2009 all switched to 0.5 mg) or a placebo once daily. In this current analysis, all patients who took either of the fingolimod doses were defined as treated. Demographic (age at baseline and sex) and clinical data were collected, including Expanded Disability Status Scale (EDSS), Paced Auditory Serial Addition Test (PASAT), Nine‐Hole Peg Test (NHPT), and Timed 25‐Foot Walk (T25FW) measured in seconds. Confirmed disability progression (CDP) at the end of the trial was defined as a binary measure to indicate the presence or the absence of clinical deterioration, as previously described [18, 19], a 1‐point increase in EDSS (from baseline to year 3) if the baseline score was ≤5.0, or a 0.5‐point increase if the baseline score was >5.0. Treatment status was unblinded after all the MRI computations were performed.

### MRI acquisitions

All patients were scanned at baseline and yearly up to the third year (end of the trial) with the following acquisitions: two‐dimensional (2D) T1‐weighted spin‐echo and 2D FLAIR, both at a resolution of 1 × 1 × 3 mm^3^. This trial was conducted across multiple centres using different MRI scanners, software versions, and field strength (1.5 T and 3 T). Overall, the study involved 148 centres in 18 countries [18].

### MRI analysis: Incident lesion detection and tissue segmentation

**FIGURE 2:**
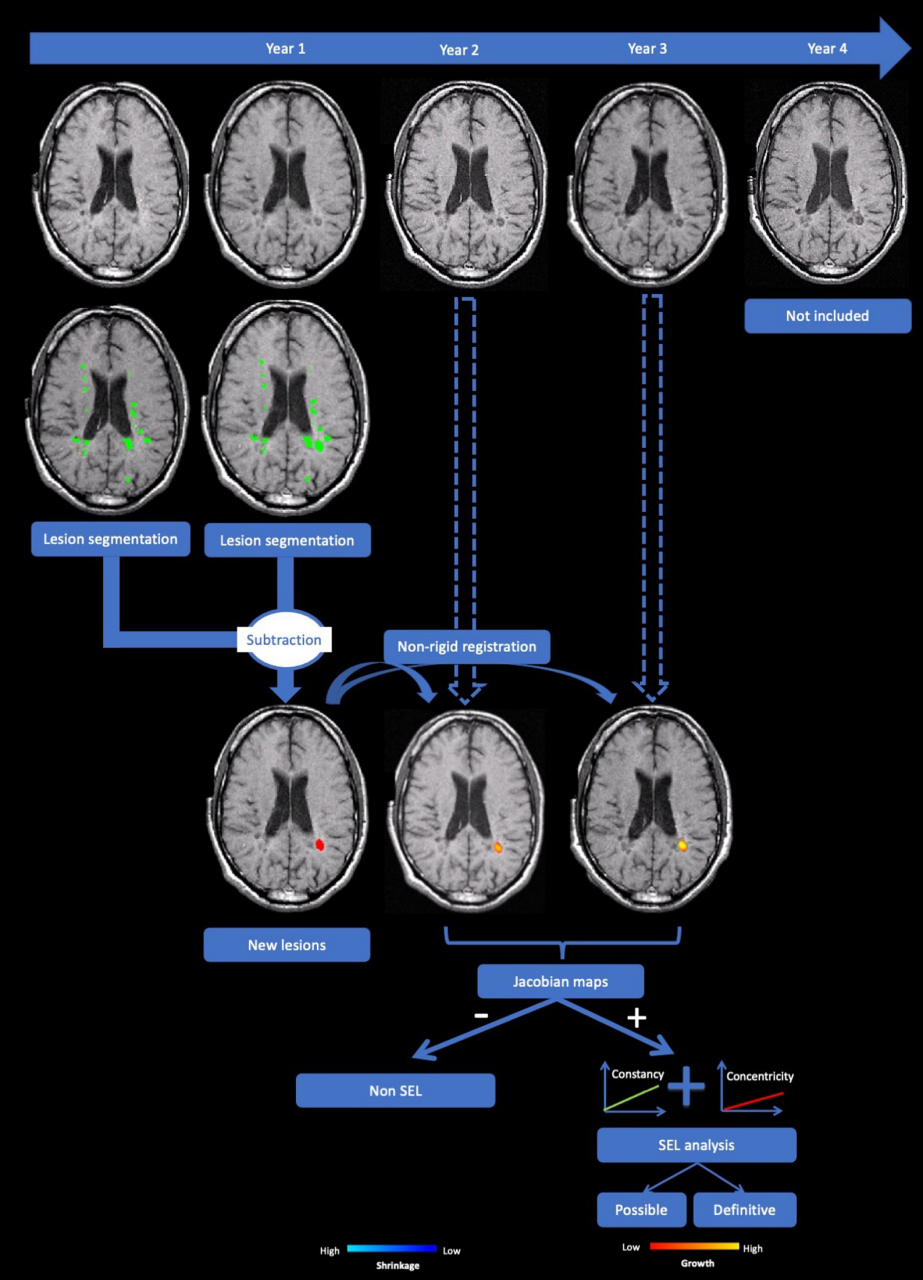
Pipeline for the evaluation of new lesions and their characterization in the slowly expanding lesion (SEL)‐based categories. From left to right, the phases of the analysis are shown. At the top left, the baseline T1‐weighted image is shown; below is the superimposed lesion segmentation at baseline, followed by the year 1 T1‐weighted image and its lesion segmentation. Below the images with segmentation masks, a subtraction image was obtained to retrieve the mask for the new lesions at year 1. The following steps include the nonlinear registration of the new lesions at year 1 with the T1‐weighted images at year 2 and year 3, including the classification using the deformation analysis into SEL (and their further subclassification into definite and possible SEL) and non‐SEL. The images of the extension phase of the trial (year 4 and beyond) were not included in this work.

Using the FLAIR images, a lesion segmentation algorithm was applied at each time point, which was based on a cascade of two 3D patchwise convolutional neural networks, as previously described [20]. The obtained cross‐sectional lesion segmentations were automatically assessed longitudinally to identify and label new (incident) lesions at year 1. Incident lesion masks were manually quality checked by three experienced raters (A.C., Z.M., and W.H.). At the patient level, the sum of individual lesion counts and volumes were calculated (Figure 2).

To obtain the global and regional brain volumes, the Geodesic Information Flow (GIF) [21] algorithm was used. Lesion‐filling was performed using a multi‐time‐point patch‐based method [22] to avoid segmentation bias. We used an in‐house version of SIENA (Structural Image Evaluation Using Normalization of Atrophy) [23] that computes the scaling factor (the difference in size between the MNI atlas brain and each subject brain). We used the scaling factor to normalize the volumes obtained by GIF to compute the following global brain and regional brain measures: normalized brain volume; normalized white matter, normalized cortical grey matter, and normalized deep grey matter volumes. The percentage brain volume change (PBVC) from baseline to year 3, as a measure of brain atrophy, was also computed.

### MRI volumetric deformation analysis

The active PPMS cohort underwent MRI volumetric deformation analysis. Deformation maps were computed using nonlinear registration by applying NiftyReg (https://github.com/KCL‐BMEIS/NiftyReg) to T1‐weighted scans across time points up to year 3, and the Jacobian expansion value (across all the study intervals) was computed at the lesion level. Incident new lesions were classified into three SEL‐based categories; definite SELs were identified based on positive *Z*‐scores of constancy (least square linear fit of lesion‐level mean Jacobian expansion value from baseline to last follow‐up) and concentricity of their expansion (least square linear fit of mean Jacobian expansion value calculated among concentric sublesion bands from the centre to the periphery), possible SELs were identified based on a positive Jacobian expansion only, and all other lesions were classified as non‐SELs [11]. The mean Jacobian expansion was determined at the lesion level and then analysed according to the SEL type. Lesion probability maps (LPMs) were obtained separately for definite, possible, and non‐SELs after registering all subjects to a common MNI anatomical atlas. MRI markers including count and volume of baseline lesions, new lesions at year 1, normalized brain and regional brain volumes, and PBVC were calculated for all the participants, and the differences between ≥1 new lesions PPMS versus no new lesions PPMS groups were analysed.

### Statistical analysis

For the statistical analysis, Stata version 16 (StataCorp) was used and the significance was reported at *p* < 0.05. R was used for graphical displays. The demographic data (sex and age at screening) and the clinical characteristics (i.e., EDSS at screening and at the final time point, and the EDSS change as the difference between the two) were analysed for all patients not included in the analysis (*n* = 646), ≥1 new lesions PPMS (*n* = 170), and no new lesions PPMS (*n* = 154). No imputation of missing data was conducted due to the nature of retrospective analysis.

At the lesion level, a descriptive analysis was performed for the population of new lesions divided into the SEL‐based types, defined as the three groups of lesions as detected by the deformation analysis: definite SEL, possible SEL, and non‐SEL. Lesion counts and volumes were analysed at the patient level calculating the sum of the number and volume of the SEL‐based types. The Wilcoxon signed‐rank test assessed the difference between the counts of the different types of lesions assessed, and a *t*‐test or a linear model was used for the normally distributed variables.

Mixed‐effect regression modeling was used to assess the association between within‐patient measures of SELs and clinical measures, including EDSS, NHPT, T25FW, and PASAT, while adjusting for age at screening, sex, baseline total T2 volume, and PBVC. In each model, we included the interaction term between each metric of interest (i.e., Jacobian expansion values, counts, and volumes of subsets of new lesions defined as definite SEL, possible SEL, and non‐SEL) and the follow‐up time, and the patient identification number and the centre were included as random effects. The coefficients of each interaction term are reported (*β*), including the 95% confidence intervals (CIs). Multiple linear and logistic regressions were applied to investigate the difference in all the MRI measures assessed between the treated and placebo subgroups of patients (as a categorical variable) and the risk of CDP calculated using within‐patient counts or log‐volumes of SELs, while adjusting for age at screening and sex. The odds ratios and *p*‐values are reported in the results.

## RESULTS

### Clinical and demographic characteristics

Demographic and clinical data and radiological parameters of the PPMS patients with ≥1 new lesions (*n* = 170), PPMS patients with no new lesions (*n* = 154), and not included (*n* = 646) PPMS groups are presented in Table 1. Of the patients included in the analysis, 52% belonged to the PPMS ≥1 new lesions group. No significant differences were found in demographic features (age and sex) and the percentage of treated patients between the PPMS patients with ≥1 new lesions, PPMS patients with no new lesions, and not included cohorts.

**TABLE 1:** Demographic and clinical characteristics.

Characteristic	PPMS, ≥1 new lesions, *n* = 170	PPMS, no new lesions, *n* = 154	Not included in the analysis, *n* = 646
Female, *n* (%)	95 (56%)	72 (47%)	302 (47%)
Age at screening, years, median (range)	48 (27 to 64)	48 (24 to 66)	49 (24 to 67)
EDSS median (range)			
At screening	4.0 (3.0 to 6.5)	4.5 (3.0 to 6.5)	4.5 (2.5 to 6.5)
At year 3	5.0 (2.0 to 8.0)	5.0 (2.0 to 8.0)	6.0 (0 to 10.0)
EDSS change, median (range)^a^	0.50 (−2.0 to 4.5)	0.50 (−2.5 to 3.0)	0.50 (−4.0 to 4.0)
NHPT, s, median (range)			
At screening	25.1 (17.2 to 68.0)	26.1 (17.3 to 105.8)	25.8 (14.2 to 189.8)
At year 3	26.3 (14.3 to 157.0)	25.4 (15.2 to 198.5)	26.2 (14.9 to 158.2)
T25FW, s, median (range)			
At screening	6.8 (3.6 to 180.7)	6.9 (3.4 to 30.0)	7.2 (3.2 to 38.4)
At year 3	8.0 (3.4 to 81.5)	8.4 (2.9 to 216.9)	8.8 (2.7 to 140.0)
PASAT score, median (range)			
At screening	44.0 (13.3 to 60.0)	47.0 (10.0 to 60.0)	42.0 (0 to 60.0)
At year 3	54.5 (2.0 to 60.0)	55.0 (2.0 to 60.0)	53.0 (0 to 60.0)
With CDP, *n* (%)^b^	71 (42%)	61 (40%)	198 (31%)
On Fingolimod, *n* (%)	90 (53%)	77 (50%)	316 (49%)

Abbreviations: CDP, confirmed disability progression; EDSS, Expanded Disability Status Scale; NHPT, Nine‐Hole Peg Test; PASAT, Paced Auditory Serial Addition Test; PPMS, primary progressive multiple sclerosis; T25FW, Timed 25‐Foot Walk.

^a^ EDSS change defined as the difference between EDSS at end of the trial (year 3) and EDSS at screening.

^b^ CDP defined as a 1‐point increase in EDSS if the score at screening was ≤5.0, or a 0.5‐point increase if the score at screening was >5.0.

In PPMS patients with ≥1 new lesions, median EDSS, median T25FW, and median PASAT significantly progressed between screening and final follow‐up (Wilcoxon signed‐rank test, *p* < 0.001 for all three variables). In all the groups of patients, the median NHPT score did not increase from screening to final follow‐up (Wilcoxon signed‐rank test, *p* = 0.166).

At screening, the PPMS patients with ≥1 new lesions exhibited a lower median EDSS (Wilcoxon signed‐rank test, *p* = 0.002) and a lower median NHPT than all the other groups (Wilcoxon signed‐rank test, *p* = 0.011). All the clinical measures were not significantly different between the three groups at the end of the follow‐up (year 3). A lower proportion of patients in the group not included in the analysis reached CDP (chi‐squared test, *p* = 0.008).

### MRI characteristics

Table 2 presents the conventional MRI measures computed. In the PPMS patients with ≥1 new lesions, we found a median of 2 new lesions (range = 1–31). At screening, the median total lesion count was significantly higher in PPMS with ≥1 new lesions (median = 27 and 22, respectively, *p* = 0.007). Similarly, the mean total lesion volume at screening was significantly higher in PPMS patients with ≥1 new lesions compared to the other group with no new lesions (8.1 vs. 5.6 mL, *p* < 0.001). All brain and regional volumes were not different between the groups, except for PBVC, which declined more rapidly in PPMS with ≥1 new lesions (−0.63% vs. −0.28% per year, *p* = 0.032), indicating a higher rate of brain atrophy.

**TABLE 2:** MRI conventional measures in PPMS patients with ≥1 new lesions versus PPMS with no new lesions.

Measure	PPMS, ≥1 new lesions, *n* = 170	PPMS, no new lesions, *n* = 154	*p* ^a^
Lesion count at screening, *n*, median (IQR)	27 (19–36)	22 (16–31)	0.008^b^
Lesion volume at screening, mL, median (IQR)	8.1 (4.6–16.7)	5.6 (2.9–10.2)	<0.001^b^
New lesion count at year 1, *n*, median (IQR)	2 (2–5)	0 (0)	‐
New lesion volume at year 1, mL, median (IQR)	0.10 (0.05–0.23)	0 (0)	‐
NBV, mL, mean (SD)			
At screening	1472.4 (84.3)	1460.8 (84.4)	0.217
At year 3	1453.8 (84.4)	1443.7 (86.4)	0.322
CGM volume, mL, mean (SD)			
At screening	768.4 (52.8)	759.4 (51.7)	0.125
At year 3	752.8 (51.8)	746.3 (49.8)	0.280
DGM volume, mean (SD)			
At screening	45.8 (3.3)	46.2 (3.5)	0.203
At year 3	45.4 (3.5)	45.6 (3.5)	0.718
DGM change, mL, mean (SD)	−0.20 (1.7)	−0.1 (1.9)	0.740
PBVC screening, year 3, mean (SD)	−0.63% (1.43)	−0.28% (1.32)	0.032^b^

Abbreviations: CGM, cortical grey matter; DGM, deep grey matter; IQR, interquartile range; MRI, magnetic resonance imaging; NBV, normalized brain volume; PBVC, percent brain volume change; PPMS, primary progressive multiple sclerosis.

^a^ Univariate linear regression, adjusted for age and sex.

^b^ *p* < 0.05.

### Descriptive analysis of new lesions and SEL‐based categories

#### Descriptive analysis at the lesion level

**FIGURE 3:**
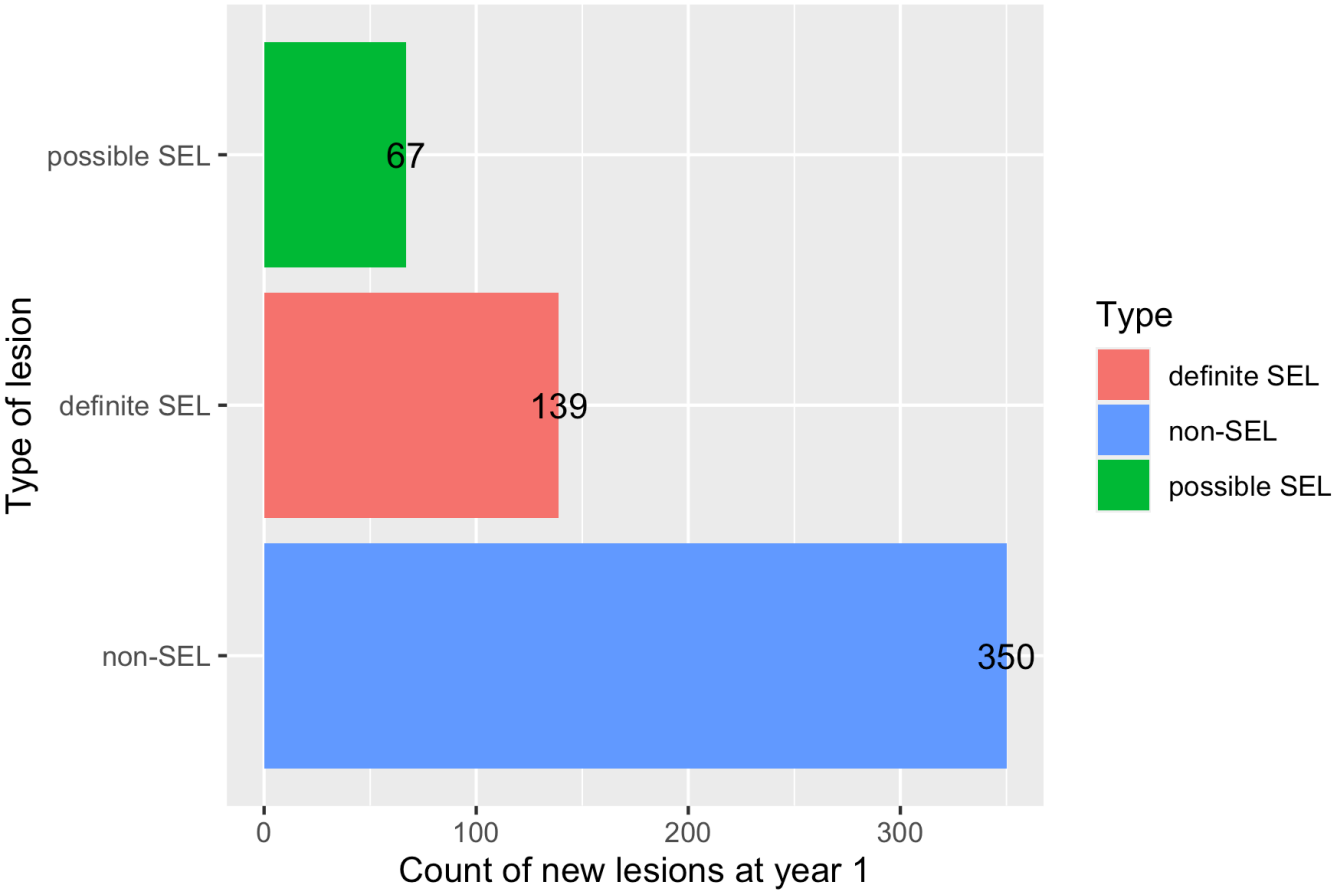
Bar plot showing the distribution of the new lesions (year 1) by type. The bar chart shows the total counts of the new lesions retrieved by subtraction (at year 1), divided in the categories as obtained with the slowly expanding lesion (SEL) algorithm.

In the PPMS patients with ≥1 new lesions (*n* = 170), 556 new lesions were identified at year 1, which had a mean volume of 0.10 mL (SD = 0.24). After applying the SEL detection algorithm, 67 definite SELs and 139 possible SELs were found. The count, volume, and proportion of lesions for each category were computed (Table S1). A bar chart showing the lesion counts divided by SEL‐based types of lesions (i.e., definite SEL, possible SEL, or non‐SEL) is presented in Figure 3.

Among the new lesions (*n* = 556), the mean Jacobian expansion value was 0.05 (SD = 0.04) from years 1 to 2 and 0.02 (SD = 0.06) from years 2 to 3. Table 3 shows the Jacobian values computed for each SEL‐based lesion type. The mean Jacobian expansion value of years 1–3 was significantly higher in the definite SELs compared to all the other lesion types (adjusted difference with the possible SEL = −0.03, 95% CI = −0.05 to −0.01, *p* = 0.002; with the non‐SELs = −0.12, 95% CI = −0.13 to −0.10, *p* < 0.001).

**TABLE 3:** Jacobian expansion values at the lesion level according to the SEL‐based categories.

Lesion type (count, % of new lesions)	Jacobian expansion value, year 1–2, mean (95% CI)	Jacobian expansion value, year 2–3, mean (95% CI)	Jacobian expansion value, year 1–3, mean (95% CI)
Non‐SEL (*n* = 350/556, 76%)	−0.06 (−0.08 to −0.05)	−0.04 (−0.06 to −0.03)	−0.06 (−0.07 to 0.05)
Possible SEL (*n* = 67/556, 9%)	0.03 (0.01 to 0.04)	−0.01 (−0.02 to 0.01)	0.03 (0.01 to 0.04)
Definite SEL (*n* = 139/556, 15%)	0.06 (0.04 to 0.07)	0.05 (0.04 to 0.06)	0.06 (0.04 to 0.07)

Abbreviations: CI, confidence interval; SEL, slowly expanding lesion.

#### Descriptive analysis of SELs at the patient level

**FIGURE 4:**
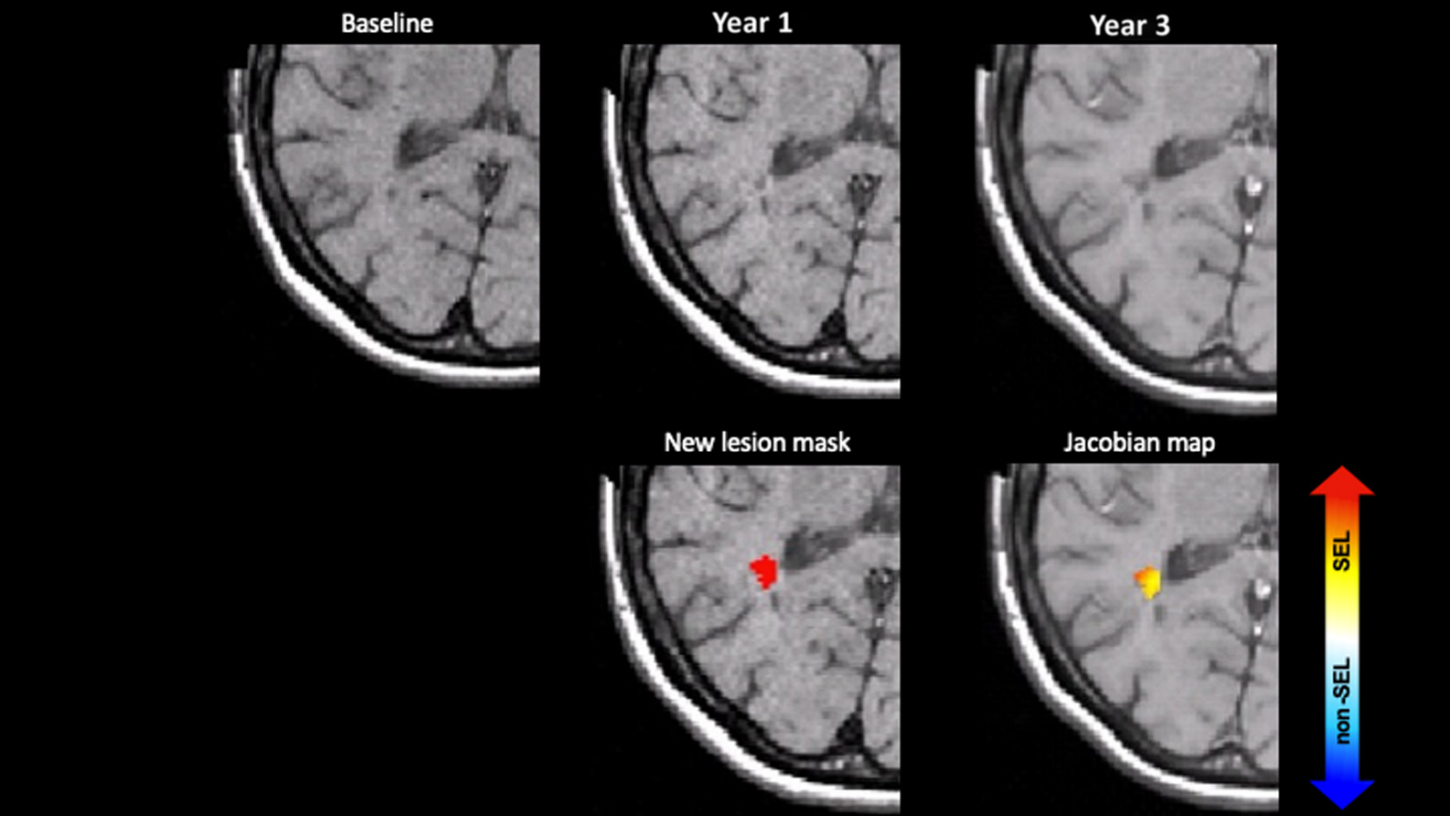
Example of a new lesion corresponding to a slowly expanding lesion (SEL). In the first row, the images from left to right are baseline, year 1, and year 2 T1‐weighted scans from a subject enrolled in the study. In the bottom row, from left to right, the mask of a new lesion at year 1 is superimposed on the corresponding T1‐weighted scan and the deformation map shows that the new lesion corresponds to an SEL.

Table 4 presents, at the patient level, the lesion count and volume, including the percentage of the total volume of the new lesions, according to the SEL‐based category. For a median count of 2 (and a mean of 3.3) new lesions per patient, 9% were definite SELs and 15% were possible SELs. An example of a patient with a new lesion detected and classified as a definite SEL is shown in Figure 4. The spatial analysis using LPMs did not show a high probability in the lesion localization according to the volumetric type (Figure S1).

**TABLE 4:** Patient‐level descriptive analysis of new lesions and the SEL categories.

Lesion type	Count, *n*, mean (SD)	Volume, mL, mean (SD)	New lesion volume, %
New lesions	3.3 (4.0)	0.33 (1.50)	‐
SEL‐based category			
Non‐SEL	2.1 (3.1)	0.25 (1.45)	76%
Possible SEL	0.4 (0.9)	0.03 (0.13)	9%
Definite SEL	0.8 (1.2)	0.05 (0.10)	15%

Abbreviation: SEL, slowly expanding lesion.

### Cross‐sectional and longitudinal associations between the radiological and clinical measures

Cross‐sectional associations between the mean Jacobian expansion values and other MRI measures (SEL‐based lesion count and regional brain volumes) and clinical baseline scores were assessed. No significant associations were found between any of the explored measures. The longitudinal analysis (mixed‐effect models; Table 5) evaluated the association between clinical scores over time. An increase in EDSS score was associated with higher definite SEL volume and count (*β* = 1.7, 95% CI = 1.0–2.4, *p* < 0.001 and *β* = 0.03, 95% CI = 0.01–0.06, *p* = 0.003), as well as higher possible SEL volume and count (*β* = 1.5, 95% CI = 0.8–2.3, *p* < 0.001 and *β* = 0.06, 95% CI = 0.03–0.09, *p* < 0.001). A longer time to complete the NHPT was associated with a higher definite SEL count, whereas a longer time to complete T25FW was associated with higher possible SEL count and volume. Using logistic regressions, the risk of patients reaching CDP was associated with increased definite SEL volume (*β* = 81.9, 95% CI = 1.6–4163.9, *p* = 0.028).

**TABLE 5:** Mixed‐effect regression models to investigate association between clinical scores and mean Jacobian expansion value and SEL‐based volume and count.

Interaction term, *β* or OR (95% CI), *p* ^a^	Outcome variable				
	EDSS	NHPT	T25FW	PASAT	CDP
Mean Jacobian expansion value	0.03 (−0.58 to 0.64), *p* = 0.925	0.91 (−13.07 to 14.90), *p* = 0.898	3.45 (−9.38, 5.93), *p* = 0.699	10.00 (−6.67, 26.69), *p* = 0.240	0.01 (0.001, 28.7), *p* = 0.331
Non‐SEL volume in mL	−0.01 (−0.02 to 0.1), *p* = 0.575	−0.01 (−0.37 to 0.35), *p* = 0.959	0.5 (−2.1 to 3.1), *p* = 0.707	−0.01 (−2.59 to 2.58), *p* = 0.996	0.6 (0.2 to 1.5), *p* = 0.265
Possible SEL volume in mL	0.4 (0.2 to 0.6), *p* < 0.001^b^	2.5 (−1.8 to 6.7), *p* = 0.263	9.6 (0.1 to 19.1), *p* = 0.048^b^	−2.9 (−11.9 to 6.0), *p* = 0.520	14.8 (0.1 to 4382.3), *p* = 0.353
Definite SEL volume in mL	0.7 (0.4 to 0.9), *p* < 0.001^b^	5.5 (−1.2 to 12.2), *p* = 0.110	8.9 (−1.3 to 19.0), *p* = 0.086	4.3 (−6.1 to 14.6), *p* = 0.420	81.9 (1.6 to 4163.9), *p* = 0.028^b^
Non‐SEL count	0.01 (−0.01 to 0.02), *p* = 0.181	0.10 (−0.09 to 0.28), *p* = 0.295	0.04 (−0.07 to 0.14), *p* = 0.465	0.01 (−0.10 to 0.11), *p* = 0.916	0.94 (0.76 to 1.15), *p* = 0.527
Possible SEL count	0.06 (0.03 to 0.09), *p* < 0.001^b^	0.60 (−0.06 to 1.26), *p* = 0.077	0.46 (0.08 to 0.84), *p* = 0.018^b^	−0.01 (−0.38 to 0.37), *p* = 0.988	1.15 (0.75 to 1.77), *p* = 0.528
Definite SEL count	0.03 (0.01 to 0.06), *p* = 0.003^b^	0.60 (0.05 to 1.14), *p* = 0.032^b^	0.21 (−0.09 to 0.51), *p* = 0.164	0.10 (−0.22 to 0.42), *p* = 0.538	1.01 (0.77 to 1.35), *p* = 0.894

Abbreviations: CDP, confirmed disability progression; CI, confidence interval; EDSS, Expanded Disability Status Scale; NHPT, Nine‐Hole Peg Test; OR, odds ratio; PASAT, Paced Auditory Serial Addition Test; SEL, slowly expanding lesion; T25FW, Timed 25‐Foot Walk.

^a^ Mixed‐effects regression models, interaction term between the independent variable (first column) and time, adjusted for age, sex, centre, percentage brain volume change, and total new lesion volume.

^b^ *p* < 0.05.

### MRI measures and treatment effect

The differences between treated and placebo groups in MRI conventional and the SEL‐based lesion type measures are reported in Table 6. In line with the original trial data results, the median new lesion count was lower in treated patients than in placebo. Lower median definite SEL count and volume were found in treated patients compared to placebo. In multiple linear regressions, after correcting for the total new lesion volume and count, both the volume and count of definite SELs were reduced in the treated group (*β* = −0.04 mL, 95% CI = −0.07 to −0.01, *p* = 0.015 and *β* = −0.36, 95% CI = −0.67 to −0.06, *p* = 0.019, respectively). There was no significant difference in possible SELs between treated and untreated, whereas higher volume and count of non‐SELs were found in treated patients (*β* = 0.06 mL, 95% CI = 0.01–0.11, *p* = 0.039 and *β* = 0.43, 95% = 0.07–0.79, *p* = 0.019, respectively).

**TABLE 6:** Differences in SEL‐based measures between the treated and nontreated patients.

Measure	Treated, *n* = 90	Placebo, *n* = 80	*p* ^a^
Lesions at screening, *n*, median (IQR)	25 (17–36)	28 (21–36)	0.119
New lesions at year 1, *n*, median (IQR)	2 (1–4)	3 (2–6)	0.037^b^
Baseline lesion volume, mL, mean (SD)	11.74 (10.21)	11.85 (10.76)	0.993
New lesion volume at year 1, mL, mean (SD)	0.37 (1.96)	0.31 (0.70)	0.299
NBV at baseline, mL, mean (SD)	1472.5 (87.8)	1472.4 (80.7)	0.869
CGM volume at baseline, mL, mean (SD)	769.4 (56.4)	767.1 (48.8)	0.891
DGM volume at baseline, mL, mean (SD)	45.8 (3.2)	45.7 (3.4)	0.724
PBVC from baseline to year 3, mean (SD)	−0.53% (1.38)	−0.55% (1.50)	0.785
Definite SEL, *n*, median (IQR)	0 (0–1)	1 (0–1.25)	0.018^b^
Possible SEL, *n*, median (IQR)	0 (0–1)	0 (0–1)	0.136
Non‐SEL, *n*, median (IQR)	1 (1–2)	1 (1–3)	0.608
Definite SEL volume, mL, mean (SD)	0.04 (0.08)	0.08 (0.12)	0.011^b^
Possible SEL volume, mL, mean (SD)	0.02 (0.06)	0.04 (0.18)	0.296
Non‐SEL volume, mL, mean (SD)	0.31 (1.99)	0.19 (0.46)	0.741

Abbreviations: CGM, cortical grey matter; DGM, deep grey matter; IQR, interquartile range; NBV, normalized brain volume; PBVC, percent brain volume change; SEL, slowly expanding lesion.

^a^ Mann–Whitney test was used for the nonnormal (count) variables, whereas the unpaired *t*‐test was used for the (log‐transformed) normally distributed volumes.

^b^ *p* < 0.05.

## DISCUSSION

This study assessed incident (new) lesions in PPMS patients and their evolution into SELs using deformation analysis. New lesions occurred in approximately half of the patients, and 15% of those evolved into definite SELs. We observed that treatment with fingolimod was associated with a reduction of SEL volume and count (*β* = −0.04 mL, 95% CI = −0.07 to −0.01, *p* = 0.015 and *β* = −0.36, 95% CI = −0.67 to −0.06, *p* = 0.019, respectively), indicative of a reduction in chronic inflammatory activity.

In this work, we reanalysed part of the INFORMS trial data; 52% of patients (170/324) included in our longitudinal analysis were defined as PPMS patients with ≥1 new lesions, as opposed to PPMS with no new lesions. PPMS patients with ≥1 new lesions had a median of 2 new lesions per patient, which was higher compared to the initial trial analysis (20%–40% of patients had at least one new or newly enlarging T2 lesion, with a mean of 0.5 per patient) [18]. This could be related to the use of an automatic lesion detection pipeline based on FLAIR rather than PD‐T2 images (used for lesion detection in the original study) and new lesions being manually quality‐checked by different raters. According to the clinical scores revised in this analysis, PPMS with ≥1 new lesions had an overall clinically more affected status compared to PPMS with no new lesions. The reduced PBVC in PPMS with ≥1 new lesions was proof of an association between brain atrophy and a higher level of inflammatory activity, as those patients were having a differential expression at both markers (i.e., presence of new lesions and steeper decrease in PBVC) assessing active inflammation and global brain damage.

In this study, when considering the new lesions only, for each patient there was a median count of 1 definite SEL and 1 possible SEL, that is, lesions showing signs of chronic activity over the subsequent 2 years of follow‐up. Lesions classified as SELs corresponded to 24% of the new lesion volume (15% definite SELs and 9% possible SELs). In previous work looking at both new and preexisting lesions [9], the mean SEL number was 6.3 in PPMS and 4.6 in the relapsing–remitting MS cohort, with a mean time since MS symptom onset of 6.4 and 6.5 years, respectively. The deformation analysis showed that the expansion was higher in all the newly formed lesions from year 1 to year 2 (Jacobian values ranging from 0.03 up to 0.06) compared to year 2 to year 3 (Jacobian values ranging from −0.01 up to 0.05). This means that overall, the Jacobian expansion value indicated an expansion within the new lesions of the SEL types (definite and possible) from year 1 to year 3. This can be interpreted as a deceleration in chronic lesion activity or the acceleration of processes leading to lesion involution (e.g., gliosis and atrophic components [24]) as multiple effects acting simultaneously on lesion volumetric changes. On the other hand, non‐SELs, as opposed to SELs, had negative mean Jacobian values, suggesting that there is a consolidation of their volume or shrinkage, potentially due to remyelination effects that have been seen in peculiar types of plaques in pathological studies [25]. In addition, in PPMS patients with ≥1 new lesions, a higher mean brain atrophy rate was found, as assessed by a steeper reduction in PBVC compared to the PPMS patients with no new lesions cohort. This suggests that patients with more incident lesions also have greater atrophy rates and the formation of new lesions might be associated with progressive neurodegeneration, which is in line with previous studies showing a high level of brain atrophy in PPMS [26].

Higher definite and possible SEL counts were associated with greater disability progression measured using multiple clinical scales. A larger definite SEL burden was associated with worse EDSS and NHPT scores. A worse T25FW score was only associated with a larger possible SEL burden. Previously, we showed that the definite SEL type might be related to a later stage of lesion evolution, as supported by lower MTR, reflecting higher tissue damage [11, 27]. In this work, definite SELs were associated with a higher level of disability in global physical and hand functions, which usually reflects more advanced disease stages. Thus, in the present analysis, the results suggest that the distal fine mobility function impairment is associated with a higher load of chronic active lesions as measured through definite SELs. Conversely, the accumulation of possible SELs, which are thought to represent a more heterogeneous stage of lesion evolution, were found to be associated with global and lower limb disability progression. Despite those findings, when we evaluated the Jacobian expansion values, we could not find any significant association with the clinical measures. The relationship between clinical scores and new lesions developing into SELs suggests that SELs contribute to predicting disability progression, but also that there are methodological limitations in our measurement of the Jacobian expansion as a quantitative value. For example, the Jacobian measures might be affected by the selected sequence for the computation phase (i.e., T1‐weighted in this analysis), registration artefacts, and the resolution of the image, due to multiple MRI field strengths and longitudinal combinations of 1.5‐ and 3‐T images.

Regarding treatment effects, new lesions occurred more often in the placebo group. In line with those results, a lower count and volume of definite SELs were found in the treated cohort. In a linear regression model, even after accounting for new lesions, a treatment effect on SELs was still apparent, suggesting this was due to not simply reduced lesion accrual but a reduction in chronic lesion activity. Conversely, there were no differences between treated and nontreated groups regarding the measures related to possible SELs, which may reflect that this marker includes a more heterogeneous stage of lesion evolution. This would mean that treatment might be not effective in some stages of lesion evolution, and this can be dependent on the distinctive characteristics of the marker.

The effect of fingolimod on 3‐ or 6‐month CDP was not significant in the previously reported main trial analysis [18], making the interpretation of the current result somewhat ambiguous. However, this discrepancy might reflect the limitation of the use of CDP as a clinical outcome, which does not fully consider the whole disability evolution as recently described by studies focused on PIRA [28, 29].

Previous trials have observed some effects of disease‐modifying treatments on MRI measures in PPMS. For example, glatiramer acetate temporarily reduced the counts of contrast‐enhancing lesions and T2 lesion volume [30]. More recently, treatment with rituximab and ocrelizumab has also shown an effect on conventional MRI measures (new/enlarging T2 lesions) in PPMS trials [31, 32]. Moreover, ocrelizumab demonstrated an effect on the reduction of T1‐hypointense SEL volume [31] as a measure of tissue damage within areas identified as chronically active lesions. Despite those results, an overall accumulation of T1‐hypointense volume in both treated and placebo arms occurred, which indicates that other unknown pathogenetic mechanisms contribute to smouldering MS and to disability progression, despite some efficacy on markers of chronic activity reported in those studies. These previous studies did not assess MS lesions from their formation, as they evaluated only the preexisting total lesion load. In the current study evaluating the expansion of new lesions, changes in the definite SEL count and volumes (at the patient level) were able to distinguish the treated and placebo populations.

There were some limitations to our study. As this work was based on original trial data, there was heterogeneity in scan acquisitions between centres and the lesion segmentation algorithm required at least two modalities for each session, including FLAIR and T1‐weighted images, which were not available for all patients at all time points. The lesion segmentation pipeline, as with other similar automated methods [33], can misidentify hyperintense areas close to the cerebral ventricles and in the meninges as lesions. To limit these errors, image‐by‐image manual quality control was undertaken. Another limitation was the inability to evaluate the difference in Jacobian values between the new lesions in relation to the chronic (preexisting) lesions. In this post hoc analysis of the INFORMS data, we found slightly different results in terms of total and new lesion count/volumes compared to the original reports and the subgroup of patients extracted for the SEL analysis had their distinctive clinical and demographical characteristics, which could have impacted on the overall results here reported. Finally, our results regarding a positive treatment effect on SEL reduction need to be carefully interpreted, because in the previous trial analysis [18] the effect of the trial medication on 3‐ or 6‐month CDP was not significant.

In future, the automated segmentation used for the identification of newly developed lesions in MS should be applied to observational MS populations and further optimized for clinical usefulness by detecting lesions that have a higher risk of developing into SELs. Moreover, the Jacobian computation as a quantitative outcome measure should be further tested and standardized for future evaluation in trials. Combined MRI deformation and pathological studies could further elucidate the mechanism of the formation and stabilization of chronic active lesions. A refinement of the technique using other methods for precisely measuring tissue changes, like boundary shift integral, could lead to an improvement in the quantification of the Jacobian values within lesions. Determining the correlation between markers of chronic active lesions in MS, including SELs, would be beneficial for identifying a standardized MRI marker of chronic inflammatory activity in MS. Data from trials assessing different disease‐modifying treatments and using other MRI markers of chronic inflammatory activity should be analysed to support the evaluation of treatment efficacy.

In conclusion, this study has shown that on average one quarter of new lesions occurring in PPMS show evidence of chronic activity over the subsequent 2 years. Fingolimod treatment may partially reduce the risk of chronic activity independently of its effect on new lesion accrual.

## AUTHOR CONTRIBUTIONS

**Alberto Calvi:** Conceptualization; funding acquisition; writing–original draft; methodology; formal analysis; data curation; investigation; validation; project administration; visualization; software. **Zoe Mendelsohn:** Validation; writing–review & editing; formal analysis. **Weaam Hamed:** Validation; formal analysis. **Declan Chard:** Writing–review & editing; investigation; conceptualization; supervision; data curation. **Carmen Tur:** Conceptualization; investigation; formal analysis; data curation; supervision. **Jon Stutters:** Software. **David MacManus:** Software; resources. **Baris Kanber:** Software; resources; methodology. **Claudia A. M. Gandini Wheeler‐Kingshott:** Conceptualization; project administration; supervision. **Frederik Barkhof:** Conceptualization; methodology; data curation; resources; project administration. **Ferran Prados:** Conceptualization; funding acquisition; project administration; data curation; supervision; methodology; software; formal analysis; validation; writing–review & editing; resources.

## CONFLICT OF INTEREST STATEMENT

We are grateful to all the IPMSA investigators who have contributed trial data to this study as part of EPITOME: Enhancing Power of Intervention Trials Through Optimized MRI Endpoints network (see the list of investigators in the Appendix S1). A. Calvi is supported by the ECTRIMS post‐doctoral training fellowship (2022), previously received a UK MS Society PhD studentship (2020), a Guarantors of Brain “Entry” clinical fellowship (2019), and an ECTRIMS‐MAGNIMS fellowship (2018). He has received travel support from the UK MS society, ECTRIMS and NAIMS. Z. Mendelsohn and B. Kanber are supported by the National Institute for Health and Research (NIHR) Biomedical Research Centre (BRC) initiative at University College London Hospitals (UCLH). W. Hamed, J Stutters have nothing to disclose in relation to this study. D. Chard is a consultant for Hoffmann‐La Roche. In the last three years he has been a consultant for Biogen, has received research funding from Hoffmann‐La Roche, the International Progressive MS Alliance, the MS Society, the Medical Research Council, and the NIHR UCLH Biomedical Research Centre, and a speaker's honorarium from Novartis. He co‐supervises a clinical fellowship at the National Hospital for Neurology and Neurosurgery, London, which is supported by Merck. C. Tur has received 2021 Merck's Award for the Investigation in Multiple Sclerosis, Junior Leader La Caixa Fellowship in 2020, ECTRIMS Post‐doctoral Research Fellowship in 2015; honoraria and support for travelling from Merck Serono, Sanofi, Roche, TEVA Pharmaceuticals, Novartis, Biogen, Bayer, Ismar Healthcare. F. Barkhof is supported by the NIHR BRC initiative at UCLH, and he serves on the steering committee, or he is iDMC member for Biogen, Merck, Roche, EISAI and Prothena. Consultant for Roche, Biogen, Merck, IXICO, Jansen, Combinostics. Research agreements with Merck, Biogen, GE Healthcare, Roche. C. Gandini Wheeler‐Kingshott has received funding from the MS Society (#77), Wings for Life (#169111), Horizon2020 (Human Brain Project SGA3, Specific Grant Agreement No. 945539), BRC (#BRC704/CAP/CGW), MRC (#MR/S026088/1), Ataxia UK. F. Prados received a Guarantors of Brain fellowship 2017‐2020 and is supported by NIHR BRC initiative at UCLH. F. Barkhof, D. Mac Manus and C. Gandini Wheeler‐Kingshott declare competing interests as share‐holders in Queen Square Analytics LTD.

## Supporting information


APPENDIX S1

## Data Availability

Data from patients are controlled by pharmaceutical companies and therefore are not publicly available. Request to access data should be forwarded to data controllers via the corresponding author. Processed data can be requested by qualified investigators from the corresponding author.
