# Supplementary material for: Treatment reduces the incidence of newly appearing multiple sclerosis lesions evolving into chronic active, slowly expanding lesions: A retrospective analysis
Source: Eur J Neurol. 2023 Oct 12;31(1):e16092. doi: 10.1111/ene.16092 (PMC11236028; doi:10.1111/ene.16092)
Supplement: Supplementary file 1 — APPENDIX S1 [file ENE-31-e16092-s001.docx]

**SUPPLEMENTARY TABLES**

**Supplementary Table 1**. Descriptive analysis of the SEL-derived lesion types and volumes

| **Lesion type** | | **Total lesions** | **Percentage of the new lesions (%)** | **Mean volume, ml (SD)** |
| --- | --- | --- | --- | --- |
| New lesion | | 556 | 100% | 0.10 (0.24) |
| Type of lesion | Non-SEL | 350 | 63% | 0.12 (0.28) |
|  | Possible SEL | 67 | 12% | 0.07 (0.10) |
|  | Definite SEL | 139 | 25% | 0.07 (0.07) |

Abbreviations: SEL=slowly expanding lesions

**Supplementary Figure 1**. Spatial analysis of the localisation of new lesions according to SEL designation status


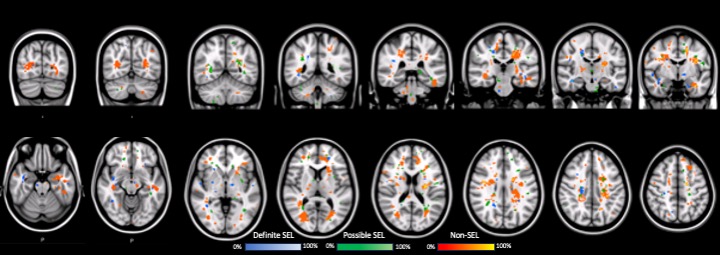


In the top row coronal views and in the bottom row the corresponding axial lesion probability maps for definite SEL, possible SEL and non-SELs. The colour bars indicate the lesion type and probability percentage (from 0 to 100%). Abbreviations: SEL= slowly-expanding lesion.

**International Progressive MS Alliance (PMSA) Investigators of the network**

| NAME | Institution |
| --- | --- |
| Douglas L Arnold | McConnell Brain Imaging Centre,  Montreal Neurological Institute, McGill  University, Montreal, Quebec, Canada |
| Sridar Narayanan | McConnell Brain Imaging Centre,  Montreal Neurological Institute, McGill  University, Montreal, Quebec, Canada |
| Frederik Barkhof | Queen Square Multiple Sclerosis  Centre, Department of  Neuroinflammation, UCL Queen Square  Institute of Neurology, Faculty of Brain  Sciences, University College London,  WC1B5EH, UK |
| Olga Ciccarelli | Queen Square Multiple Sclerosis  Centre, Department of  Neuroinflammation, UCL Queen Square  Institute of Neurology, Faculty of Brain  Sciences, University College London,  WC1B5EH, UK |
| Declan Chard | Queen Square Multiple Sclerosis  Centre, Department of  Neuroinflammation, UCL Queen Square  Institute of Neurology, Faculty of Brain  Sciences, University College London,  WC1B5EH, UK |
| Louis Collins | McConnell Brain Imaging Centre,  Montreal Neurological Institute, McGill  University, Montreal, Quebec, Canada |
| Tal Arbel | McConnell Brain Imaging Centre,  Montreal Neurological Institute, McGill  University, Montreal, Quebec, Canada |
| Charles R.G Guttman | Center for Neurological Imaging,  Brigham and Women’s Hospital,  Harvard Medical School,  Massachusetts, USA |
| Jerry S Wolinsky | McGovern Medical School, The  University of Texas Health Science  Center at Houston (UTHealth), Houston,  Texas, USA |
| Garry R Cutter | University of Alabama at Birmingham  School of Public Health, USA |
| Nicola De Stefano | University of Siena, Italy |
| Maria Pia Sormani | University of Genoa, Italy |
| Ludwig Kappos | University Hospital Basel, Switzerland |
| Jack H Simon | Oregon Health and Sciences University,  Portland Veterans Affairs Medical  Center, Oregon, USA |
| Jeremy Chataway | Queen Square Multiple Sclerosis  Centre, Department of  Neuroinflammation, UCL Queen Square  Institute of Neurology, Faculty of Brain  Sciences, University College London,  WC1B5EH, UK |
| Raj Kapoor | Queen Square Multiple Sclerosis  Centre, Department of  Neuroinflammation, UCL Queen Square  Institute of Neurology, Faculty of Brain Sciences |
| Howard L. Weiner (CLIMB Investigator) | Brigham and Women’s Hospital, Ann  Romney Center for Neurologic  Diseases, Department of Neurology,  Boston, MA, 02115 |
| Tanuja Chitnis (CLIMB Investigator) | Brigham and Women’s Hospital, Ann  Romney Center for Neurologic  Diseases, Department of Neurology,  Boston, MA, 02115 |
| Rohit Bakshi (CLIMB Investigator) | Brigham and Women’s Hospital, Ann  Romney Center for Neurologic  Diseases, Department of Neurology,  Boston, MA, 02115 |
